# The World’s Columbian Exposition

**Published:** 1892-06

**Authors:** 


### EDITORIAL.

### The World's Columbian Exposition.

The scope of the Eposition has grown as the work has progressed; more than seven thousand six hundred car loads of building material have already been received on the Exposition grounds. The preliminary estimates of the cost of the work are entirely inadequate to such an Exposition as the people of the United States expect to be produced under national auspices. The classification comprises exhibits on an enormous scale in departments that have heretofore either been ignored or lightly treated in great expositions, or made the subject of special expositions at great expense. The area embodied in the Exposition grounds will be nearly three times that of the greatest Exposition heretofore held. The enthusiasm and interest of the people of the United States has kept pace with the demand for increased appropriations, and many of the States and Territories have recently added largely to their former appropriations, which now aggregate three million, one hundred and eighty thousand dollars; Maryland and New York, respectively, have voted sixty thousand and three hundred thousand dollars; New Jersey has increased its appropriation of twenty thousand by fifty thousand dollars; Iowa, its fifty thousand by one hundred and twenty-five thousand dollars; Massachusetts and other States have also added largely to their former appropriations. The World's Fair Board for Kansas is promoting a plan whereby it is expected that the expense of erecting the Exposition building for that State will be borne by school pupils. The proposition is to have all of the schools in the State observe a "World's Fair Day," by holding an entertainment with music, recitations, tableaux, etc., to which a small entrance fee will be charged. The proceeds will be sufficient to pay for the State building. Over the main entrance to the structure, it is proposed to have the words: "Erected by the School Children of Kansas."

The World's Fair appropriation by foreign countries, as far as reported, aggregates more than four million, five hundred thousand dollars. Exhibits from foreign ports are already beginning to arrive at New York in considerable numbers, and the Secretary of the Treasury has instructed the collectors of Customs at all United States ports, that the transportation of articles intended for exhibit at the Exposition must be facilitated in preference to all other importations.

Chief Willard Smith, of the Department of Transportation, is arranging for a large number of interesting exhibits; recently he has been paying special attention to the marine section. In it will be models of the old frigate "Constitution;" the flag-ship of Nelson; a caravel from Spain, the exact copy of the "Santa Maria," in which Columbus made his first voyage; canoes of the native traders of the West Indies, hewn from a single tree and propelled by twenty-five paddles; models of such modern racing schooners as the America, Mayflower, Puritan and Volunteer. All sorts of steamers for river navigation, steel-screw ferry-boats, electric pinnaces, naptha launches, ketches and brigantines, sloops and barques of the Atlantic coast in 1714, rafts, arks, barges, keelboats and other craft.

From Holland an offer has been made to the Holland Society of New York, and the St. Nicholas Society of Brooklyn, to construct and present to them an exact reproduction of the Half Moon, the ship in which Henry Hudson discovered and explored the river which bears his name. The societies named have accepted the offer, and are planning to fit up the ship as a club house, and to take it to Chicago, both to be exhibited and to be occupied by their members during the Exposition.

One of the attractive features of the Australian exhibit will be the tree ferns from Sidney, New South Wales. These have always been a popular exhibit at London Expositions. They vary in height from eight to fourteen feet.

An immense wooden box, bound in iron, was recently found at Helsinfors, in Finland, by workmen engaged in excavating in the cellar of an old house. Upon opening the box, the men found that it contained a large parchment and a quantity of pieces of iron of odd shapes. Being unable to make out the contents of the parchment, they carried it to Mr. Rizeff, the nearest magistrate, who found that it was written by Father Suger, at one time minister to Louis VII of France. It was an elaborately written treatise upon the use of steam as a motive power, and further examination revealed that the bits of iron were numbered parts of a rudimental but complete steam engine. It is proposed to fit the parts together, and to exhibit this pioneer steam engine at the Exposition.

